# Application Scope and Limitations of TADDOL-Derived Chiral Ammonium Salt Phase-Transfer Catalysts

**DOI:** 10.3390/molecules18044357

**Published:** 2013-04-12

**Authors:** Guddeangadi N. Gururaja, Richard Herchl, Antonia Pichler, Katharina Gratzer, Mario Waser

**Affiliations:** Institute of Organic Chemistry, Johannes Kepler University Linz, Altenbergerstraße 69, 4040 Linz, Austria

**Keywords:** asymmetric catalysis, tartaric acid, α-alkyation, Michael addition

## Abstract

We have recently introduced a new class of chiral ammonium salt catalysts derived from easily available TADDOLs. To get a full picture of the scope of application and limitations of our catalysts we tested them in a variety of different important transformations. We found that, although these compounds have recently shown their good potential in the asymmetric α-alkylation of glycine Schiff bases, they clearly failed when we attempted to control more reactive nucleophiles like β-keto esters. On the other hand, when using them to catalyse the addition of glycine Schiff bases to different Michael acceptors it was found necessary to carefully optimize the reaction conditions for every single substrate class, as seemingly small structural changes sometimes required the use of totally different reaction conditions. Under carefully optimized conditions enantiomeric ratios up to 91:9 could be achieved in the addition of glycine Schiff bases to acrylates, whereas acrylamides and methyl vinyl ketone gave slightly lower selectivities (up to e.r. 77:23 in these cases). Thus, together with additional studies towards the syntheses of these catalysts we have now a very detailed understanding about the scope and limitations of the synthesis sequence to access our PTCs and about the application scope of these catalysts in asymmetric transformations.

## 1. Introduction

Design, syntheses, and applications of chiral phase-transfer catalysts (PTCs) have attracted considerable interest over the last three decades [1,2,3,4,5,6]. The high potential of asymmetric phase-transfer catalysis can be attributed to several reasons (e.g., mild aqueous reaction conditions, operational simplicity, easily handled catalysts, scalability,..), making it a powerful and versatile methodology for a broad scope of different applications where other catalytic principles clearly fail. Among the different commonly employed catalytically active structural motives, chiral quaternary ammonium salts have found the most widespread applications so far [1,2,3,4,5,6]. Following the seminal reports of Wynberg [7] and a group of Merck scientists [8] employing cinchona alkaloid-derived quaternary ammonium salts for asymmetric epoxide formation [7] and methylation of a phenylindanone derivative [8], cinchona alkaloids remained the privileged source of chirality for syntheses and investigations concerning novel phase-transfer catalysts and applications thereof until the beginning of the 21st century. Pioneering work by the groups of O’Donnell [9,10], Lygo [11,12], and Corey [13,14] resulted in the development of several highly stereoselective applications using a variety of structurally carefully optimized cinchona alkaloid-based PTCs. Due to their high catalytic potential and broad application scope, catalysts based on this easily obtained naturally occurring chiral backbone still belong to the most commonly employed and most thoroughly investigated PTCs as shown in recent reports by the groups of Li Deng [15,16], Jørgensen [17,18], and others [19,20,21,22,23,24,25,26,27,28,29].

In 1999, Maruoka introduced a new designer catalyst system by using *C*_2_-symmetric binaphthyl-based chiral spiro ammonium salts [30]. These Maruoka catalysts were found to be highly effective for a variety of asymmetric transformations (e.g., Michael additions, α-amino acid syntheses, epoxidations, aldol-type reactions, isoxazoline syntheses,..), even using only minimum amounts of catalysts (<1 mol%) [4,5,30,31,32,33,34,35], thus belonging to the most powerful and versatile PTCs known to date. In addition, also Shibasaki’s tartaric acid-derived bidentate PTCs [36,37,38] and Lygo’s biphenyl-based spirocyclic catalysts [39,40] have proven their potential in different asymmetric applications.

**Figure 1:**
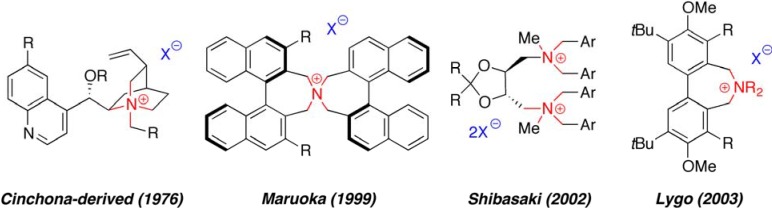
Privileged chiral ammonium salt PTCs.

However, despite more than three decades of active research in this field it is somewhat surprising that besides the already mentioned privileged catalyst structures (Figure 1) only a few other classes of chiral ammonium salt PTCs have been reported so far [41,42,43,44,45]. Despite sometimes very exhaustive and careful structure-activity based investigations and optimizations [44,45], none of these other classes has so far reached the catalytic potential and application scope of especially the cinchona-based catalysts and the Maruoka-type catalysts. 

**Scheme 1:**
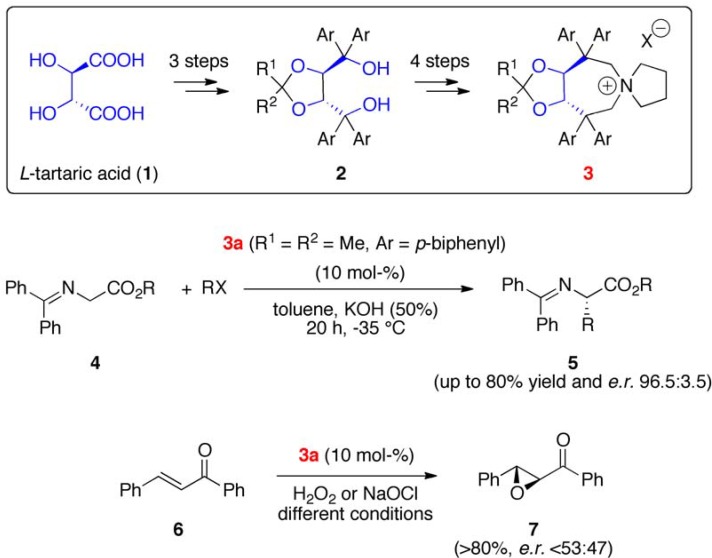
Recently described synthesis of TADDOL-derived *N*-spiro ammonium salt catalysts **3** and their performance in initial test reactions [48,49].

One of the main demands for novel catalysts is easy accessibility from readily available chiral starting materials. Among the easily available natural chiral sources, tartaric acid (**1**) has obtained a prominent position, especially due to the fact that both enantiomers are readily available in sufficient quantities. Although Shibasaki *et al.* have demonstrated the potential of tartaric acid-derived bidentate PTCs [36,37,38], others were less successful in their attempts to synthesize powerful tartaric acid-derived quaternary ammonium salt catalysts [41,42]. Based on the high potential of tartaric acid-derived easily obtainable tetraaryl-2,2-dimethyl-1,3-dioxolan-4,5-dimethanols (TADDOLs, **2**) as chiral ligands in (transition-) metal catalysis [46,47] we have recently carried out systematic investigations to use this unique structural motive for the syntheses of chiral *N*-spiroquaternary ammonium salt catalysts [48,49]. After a careful route development we were able to obtain more than 30 differently substituted *C*_1_- or *C*_2_-symmetric *N*-spiro catalysts **3**. The catalytic potential of these PTCs was initially tested for the benchmark α-alkylation of glycine Schiff base **4** and the *p*-biphenyl containing acetonide-based catalyst **3a** turned out to be the most powerful one therein, giving access to a variety of amino acid derivatives **5** in high yields and with satisfying enantioselectivities (Scheme 1). In contrast, testing this catalyst for the asymmetric epoxidation of chalcone **6** resulted in the formation of racemic **7** only [49].

To further elucidate the potential and the application scope of this novel and straightforwardly available class of catalysts we have now carried out a detailed screening of different other important test reactions. In addition further attempts to systematically modify the catalyst structures have been undertaken.

## 2. Results and Discussion

### 2.1. Late Stage Catalyst Modification

**Scheme 2:**
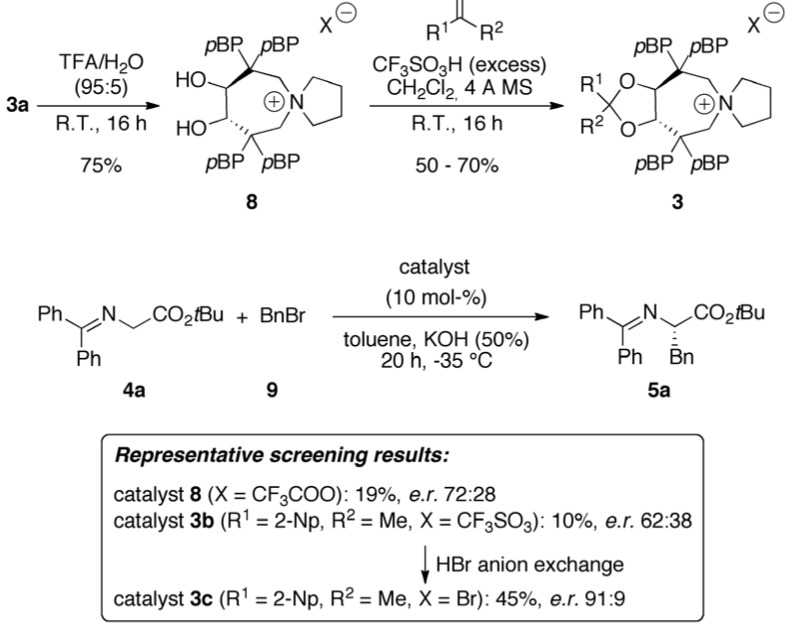
Late-stage acetal modification of **3a** to access **8** and differently substituted ammonium salts **3** and their catalytic potential.

We have recently observed that the nature of the acetal protecting group of the catalyst has a strong influence on the catalyst performance in the benchmark α-alkylation of **4** [49]. Unfortunately, our standard strategy to access these catalysts required introduction of the acetal group very early in the sequence already, making a rapid structural diversification tedious, especially as we found that, based on the nature of the acetal group, the subsequent steps sometimes proceeded significantly lower yielding (or were even not possible anymore) and purification of the final products also became difficult [49]. Thus, we targeted a late stage acetal-cleavage – acetal-formation sequence starting from readily available **3a** to access differently acetal-protected catalysts straightforwardly. Interestingly the dioxolane moiety was found to be exceptionally acid-stable and it required treatment with concentrated trifluoroacetic acid (TFA/H_2_O = 95:5) to obtain the diol **8** (Scheme 2). Initial attempts to test the free-OH containing ammonium salt **8** as a catalyst for the reaction of the glycine Schiff base *t*-butyl ester **4a** with benzylbromide (**9**) gave surprising results. First the enantioselectivity was rather low (e.r. 72:28) under the previously optimized conditions and, even more interesting, the product **5a** was only obtained in less than 20% yield. Furthermore the catalyst could not be recovered, but decomposed almost quantitatively under the basic reaction conditions. This pronounced base-sensitivity was also observed when we attempted an *O*-benzylation or *O*-methylation of **8** even using just bicarbonates as the bases, thus making syntheses of diether-derivatives of these catalysts impossible (these compounds were also not accessible using our standard procedure) [49]. In contrast, compound **8** was found to be rather acid-stable and dioxolane-formation with different ketones or aldehydes could be carried out in the presence of triflic acid. Noteworthy, these reactions only proceeded with an excess of this strong acid, whereas other strategies failed, thus giving the corresponding ammonium triflates **3** first. However, these catalysts absolutely failed in the test reaction as no turnover and only modest enantioselectivities were observed (an illustrative example using catalyst **3b** is given in Scheme 2 and similar results were obtained using other differently acetal-protected ammonium salts prepared by this strategy). As we recently observed a significant counter anion influence in this alkylation (e.g., changing Br^−^ for other halides did not affect the activity, but using BF_4_^−^ or PF_6_^−^ reduced the catalytic potential dramatically [49]), we tried different counter anion exchange methods to obtain the ammonium bromides. However, only the use of HBr allowed us to replace the triflate anion to some extent, but always accompanied with significant decomposition, which made isolation and purification by standard methods very tedious and low yielding. Testing this (not perfectly pure) catalyst **3c** an improved, but still not satisfying α-alkylation result was obtained (see Scheme 2). Noteworthy, we have recently prepared catalyst **3c** via our conventional strategy (but were not able to obtain it in sufficient yield and purity either), which showed a better catalytic potential than material obtained by the new acetal transformation - counter anion exchange method [49]. Accordingly, although this late-stage modification strategy seemed promising at first, it did not allow us to obtain the targeted catalysts in sufficient quality and with strict control of the nature of the counter anion and thus could not be readily and reliably used for stereoselective applications. 

### 2.2. Asymmetric α-Alkylation of β-Keto Esters

**Scheme 3:**
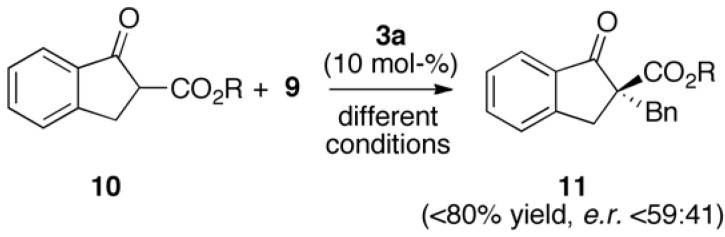
Attempted **3a**-catalysed asymmetric α-alkylation of β-keto esters **10**.

As we have recently proven the high potential of our catalysts for asymmetric α-alkylation reactions of glycine Schiff bases we also tested their applicability for the asymmetric α-alkylation of β-keto esters. As a test reaction we choose the benzylation of esters **10** under a variety of different liquid/liquid or liquid/solid phase-transfer conditions (Scheme 3). Unfortunately, after an extensive screening of a variety of different conditions and also differently substituted esters **10** we were not able to obtain the products **11** with any reasonable enantiopurity. In contrast, the non-catalysed racemic background alkylation of this highly acidic starting material was found to be the dominating reaction therein. Thus, it seems reasonable that formation of the required chiral ion pair between the ammonium salt catalyst and the enolate of **10** is too slow compared to the non-catalysed racemic background reaction, thus explaining the low enantioselectivities observed in this specific test reaction.

### 2.3. Asymmetric Michael Addition Reactions of Glycine Schiff Bases

Besides asymmetric α-alkylation reactions also the analogous Michael addition reactions have emerged as powerful applications of asymmetric PTCs in the past. To elucidate the potential of our catalysts for such transformations we investigated their use for the reaction of glycine Schiff bases **4** with a variety of different acrylates **12** next (Table 1 gives an overview of the most significant results obtained in a thorough screening of different reaction conditions, reagents, and catalysts). Initial experiments were carried out in analogy to our recent α-alkylation protocol using the standard biphenyl catalyst 3a (10 mol%) in toluene as the solvent and with aqueous KOH as the base at 0 °C (entries 1 and 2). Surprisingly, absolutely no enantioselectivity could be obtained in the addition of the *t*-butyl ester **4a** to methyl acrylate **12a**. Also the use of solid KOH (entry 3) or the use of weaker aqueous bases like K_3_PO_4_ (entry **4**) or different alkali carbonates (entries 5 and 6) gave racemic **13a** in low yields only. However, when we used an excess of solid Cs_2_CO_3_ as the base, the product **13a** was obtained in modest enantioselectivity (e.r. 66:34) and with good yield (entry 7). Reducing the reaction temperature to −20 °C gave a slightly improved enantiomeric ratio of 71:29 (the e.r. could be increased to 75:25 upon using 20 mol% of catalyst). At this point we observed that the use of recovered catalyst (after extractive workup and column chromatography) resulted in a significantly reduced enantioselectivity compared to the use of freshly prepared catalyst (entry 9 *vs.* entry **8**, this also explains why using 20 mol% of catalyst allowed us to obtain the product in higher yield and with better selectivity than using 10 mol%). As the only difference in these two cases seems to be the nature of the counter anion due to an exchange of the bromide to either carbonate or chloride (due to brine extraction) we next tested the systematically modified catalysts **3d** (with BF_4_^−^ as the counter anion) and **3e** (PF_6_^−^) (entries 10 and 11). Unfortunately, in neither case an increased selectivity could be achieved [50,51]. Noteworthy, the use of those catalysts prepared via our acetal-deprotection – protection strategy having either a trifluoroacetate or a triflate counter anion (see Scheme 2) also did not allow us to obtain the Michael product in any reasonable quantity and enantiopurity. Accordingly, to obtain reproducible and comparable results for the rest of these studies we always used freshly prepared ammonium bromide catalysts (comparable results were obtained when recovered catalyst was refluxed in acetonitrile with an excess of KBr for 2 days, thus giving the corresponding ammonium bromide again). Next, a screening of different solvents revealed mesitylene to be the best-suited one (non-aromatic solvents were found to be not suitable). Interestingly, addition of different additives was found to have no beneficial effect. For example the use of molecular sieves significantly suppressed the yield and the enantioselectivity (entry 15) whereas on the other hand addition of a proton source (as described recently to be beneficial by Lygo *et al*. [52]) also did not allow us to achieve a higher selectivity (entry 16). Unfortunately also the addition of different inorganic salts (e.g., CsBr, KBr, CsF or others) did not have any beneficial effect.

**Table 1:** Asymmetric Michael addition of glycine Schiff bases **4** to acrylates **12** catalysed by TADDOL-derived ammonium salts **3**. 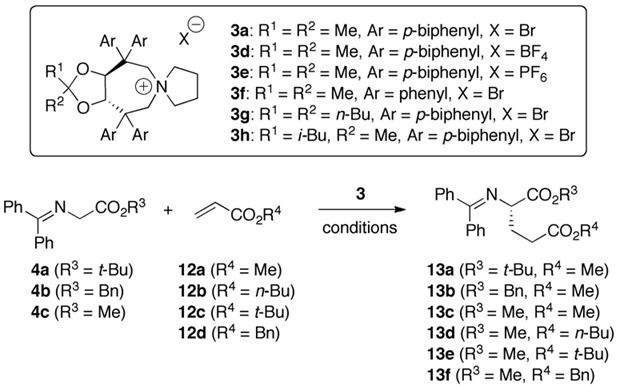

**Entry ^a^**	**Cat. (mol%)**	**4**	**12**	**Solv.**	**Base (eq.)**	**T [°C]**	**13**	**Yield ^b^ [%]**	**e.r. ^c^ (conf.) ^d^**
1	3a (10%)	4a	12a	toluene	KOH (50%) (25×)	0	13a	94	51:49 (*S*)
2	3a (10%)	4a	12a	toluene	KOH (50%) (1×)	0	13a	**88**	50:50
3	3a (10%)	4a	12a	toluene	KOH (s) (20×)	0	13a	76	50:50
4	3a (10%)	4a	12a	toluene	K_3_PO_4_ (50%) (10×)	0	13a	34	50:50
5	3a (10%)	4a	12a	toluene	K_2_CO_3_ (50%) (10×)	0	13a	1**8**	52:4**8** (*S*)
6	3a (10%)	4a	12a	toluene	Cs_2_CO_3_ (70%) (10×)	0	13a	10	50:50
7	3a (10%)	4a	12a	toluene	Cs_2_CO_3_ (s) (20×)	0	13a	73	66:34 (*S*)
**8**	3a (10%)	4a	12a	toluene	Cs_2_CO_3_ (s) (20×)	−20	13a	56	71:29 (*S*)
9 ^e^	3a (10%) ^e^	4a	12a	toluene	Cs_2_CO_3_ (s) (20×)	−20	13a	62	61:39 (*S*)
10	3d (10%)	4a	12a	toluene	Cs_2_CO_3_ (s) (20×)	−20	13a	14	62:3**8** (*S*)
11	3e (10%)	4a	12a	toluene	Cs_2_CO_3_ (s) (20×)	−20	13a	**8**2	64:36 (*S*)
12	3a (10%)	4a	12a	benzene	Cs_2_CO_3_ (s) (20×)	0	13a	72	5**8**:42 (*S*)
13	3a (10%)	4a	12a	fluorobenzene	Cs_2_CO_3_ (s) (20×)	0	13a	**8**9	54:46 (*S*)
14	3a (10%)	4a	12a	mesitylene	Cs_2_CO_3_ (s) (20×)	0	13a	74	69:31 (*S*)
15 ^f^	3a (10%)	4a	12a	mesitylene	Cs_2_CO_3_ (s) (20×)	0	13a	33	57:43 (*S*)
16 ^g^	3a (10%)	4a	12a	mesitylene	Cs_2_CO_3_ (s) (20×)	0	13a	76	51:49 (*S*)
17	3a (10%)	4b	12a	mesitylene	Cs_2_CO_3_ (s) (20×)	0	13b	66	75:25 (*S*)
1**8**	3a (10%)	4c	12a	mesitylene	Cs_2_CO_3_ (s) (20×)	0	13c	**8**1	7**8**:22
19	3a (10%)	4c	12a	mesitylene	Cs_2_CO_3_ (s) (20×)	−20	13c	35	**8**5:15
20	3a (20%)	4c	12a	mesitylene	Cs_2_CO_3_ (s) (20×)	−20	13c	71	90:10
21	3a (20%)	4c	12b	mesitylene	Cs_2_CO_3_ (s) (20×)	−20	13d	6**8**	**8**9:11
22	3a (20%)	4c	12c	mesitylene	Cs_2_CO_3_ (s) (20×)	−20	13e	n.r.	n.d.
23	3a (20%)	4c	12d	mesitylene	Cs_2_CO_3_ (s) (20×)	−20	13f	**8**1	**8**7:13
24	3f (20%)	4c	12a	mesitylene	Cs_2_CO_3_ (s) (20×)	−20	13c	51	**8**0:20
25	3g (20%)	4c	12a	mesitylene	Cs_2_CO_3_ (s) (20×)	−20	13c	56	**8**6:14
26	3h (20%)	4c	12a	mesitylene	Cs_2_CO_3_ (s) (20×)	−20	13c	68	91:9

^a^ 22 h reaction time under an Ar-atmosphere using 1.5 equiv. of the acrylate **12**; ^b^ Isolated Yield; ^c^ Determined by HPLC using a chiral stationary phase. In each case the (−)-enantiomer was the major one; ^d^ Determined by comparison of the HPLC retention time and the optical rotation with values reported in literature (**13a** [53]; **13b** [54]); ^e^ Using recovered catalyst; ^f^ Using 4Å molecular sieve as an additive; ^g^ Using mesitol as an additive.

Testing different esters **4** next, we observed a strong influence of the ester moiety (compare entries 14, 17, and 18). In contrast to our recent results obtained when we used these esters for asymmetric α-alkylation reactions were we found the *t*-butyl ester Schiff base **4a** to give by far the best yields and highest selectivities [49], the opposite tendency was observed in the present Michael addition. Herein the methyl ester Schiff base **4c** was found to be the best suited one, giving **13c** with an e.r. of 78:22 at 0 °C (entry 18). Lowering the reaction temperature resulted in an improved selectivity, albeit with a significantly lower yield (entry 19), which could be overcome by using 20 mol% catalyst instead, giving **13c** with 90:10 e.r. and in 71% yield (entry 20). Under these optimized conditions we employed different acrylates **12** next (entries 20–23). Interestingly, whereas the methyl, *n*-butyl, and benzyl esters performed similarly well, no product was obtained when we used the *t*-butyl ester 12c. Finally, to investigate the importance of the catalyst substituents we performed the reaction between methyl Schiff base **4c** and methyl acrylate **12a** in the presence of different *C*_1_ or *C*_2_-symmetric PTCs **3** (The most illustrative results are summarized in entries 24–26, Table 1). In analogy to our recent alkylation results the phenyl-based catalyst **3f** performed less selective and lower yielding than catalyst **3a** (entry 24 *vs.* entry 20). Also the *n*-butyl containing *C*_2_-symmetric catalyst **3g** was found to be less selective in both, alkylation [49] and Michael reaction (entry 25). Interestingly, the *C*_1_-symmetric catalyst **3h** performed even slightly better in the Michael addition than **3a** (entry 26 * vs.* entry 20), which is in contrast to our recent alkylation results, where this catalyst was slightly less selective than **3a** [49]. Unfortunately, no further improvement could be achieved by using any other of our recently introduced catalysts anymore. 

Having optimized the conditions for the asymmetric Michael addition of glycine Schiff bases **4** to acrylic acid esters **12** we then screened the use of other Michael acceptors like acrylamides **14** and methyl vinyl ketone (MVK, **15**) (Table 2 gives a comprehensive overview about the results obtained with catalyst **3a**). Use of acrylamides as acceptors in PT-catalysed Michael reactions has only sparingly been reported in the past [40,55] and, interestingly enough, these seemingly subtle changes in the Michael acceptor resulted in a totally different behaviour in our test reaction with glycine Schiff bases **4**. Using the conditions that have been optimized for the addition to acrylates **12** first, we found that only small amounts of racemic product **16a** could be obtained when reacting Schiff base **4a** with the acrylamide **14a** (entries 1 and 2). Also the use of other weaker solid bases did not give any product (entries 3 and 4 give just two examples of the tested ones) whereas the use of solid KOH (entry 5) gave a significant conversion, but almost no enantioselectivity (the same was observed using other solid hydroxide bases). Interestingly, when we used aqueous KOH, the product was obtained in reasonable yield and with a low enantiomeric ratio of 65:35 (entry 6). Similar results were obtained using other aqueous alkali hydroxide bases (entries 7 and 8) with RbOH being the best-suited one (e.r. 69:31 at 0 °C). Noteworthy, the observed tendency that aqueous hydroxide bases perform better than solid bases is in sharp contrast to our observations with the Michael additions to acrylates (Table 1) where aqueous bases clearly failed whereas solid ones performed superior. Further testing of different solvents and different conditions showed that for this reaction toluene is the superior solvent. Finally running the reaction for 2 days the product **16a** was obtained in reasonable 65% yield and with a modest enantiomeric ratio of 75:25 (no further improvement was possible also due to the low reaction rate of this reaction at reduced temperature). Unfortunately, using the *N*,*N*-diphenyl acrylamide **14b** as an acceptor the enantioselectivity dropped significantly again (entries 11 and 12) and testing secondary amides (not in the table) no product was formed, thus illustrating that Michael addition to acrylamides under asymmetric phase-transfer conditions is a rather challenging transformation. Using MVK as the acceptor both liquid/liquid and liquid/solid conditions gave the product, but the milder liquid/liquid conditions were found to be slightly better suited to give the Michael product **17** with a modest enantiomeric ratio of 77:23 (entry 14).

**Table 2:** Asymmetric Michael addition of glycine Schiff bases **4** to different Michael acceptors catalysed by **3a**. 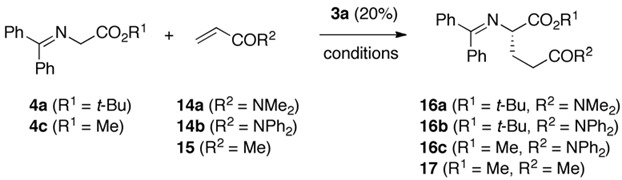

**Entry**	**4**	**Acceptor (eq.)**	**Solv.**	**Base (eq.)**	**T [°C]**	**t [h]**	**Prod.**	**Yield ^a^ [%]**	**e.r. ^b^**
1	4a	14a (2×)	mesitylene	Cs_2_CO_3_ (s) (20×)	0	20	16a	32	50:50
2	4a	14a (2×)	mesitylene	Cs_2_CO_3_ (s) (1×)	0	20	16a	11	50:50
3	4a	14a (2×)	toluene	K_2_CO_3_ (s) (1×)	0	20	16a	n.r.	n.d.
4	4a	14a (2×)	toluene	K_2_HPO_4_ (s) (1×)	0	20	16a	n.r.	n.d.
5	4a	14a (2×)	toluene	KOH (s) (1×)	0	20	16a	55	56:44
6	4a	14a (2×)	toluene	KOH (50%) (25×)	0	20	16a	62	65:35
7	4a	14a (2×)	toluene	CsOH (50%) (25×)	0	20	16a	55	66:34
8	4a	14a (2×)	toluene	RbOH (50%) (25×)	0	20	16a	69	69:31
9	4a	14a (2×)	mesitylene	RbOH (50%) (25×)	0	20	16a	62	60:40
10	4a	14a (2×)	toluene	RbOH (50%) (25×)	−20	48	16a	65	75:25
11	4a	14b (2×)	toluene	RbOH (50%) (25×)	−20	48	16b	81	60:40
12	4c	14b (2×)	toluene	RbOH (50%) (25×)	−20	48	16c	64	57:43
13	4c	15 (2×)	toluene	RbOH (50%) (25×)	−20	48	17	65	67:33 (*S*) ^c^
14	4c	15 (1.5×)	mesitylene	Cs_2_CO_3_ (s) (20×)	−20	48	17	97	77:23 (*S*) ^c^

^a^ Isolated Yield; ^b^ Determined by HPLC using a chiral stationary phase; ^c^ Determined by comparison of the optical rotation with literature value [56].

## 3. Experimental

### 3.1. General

^1^H- and ^13^C-NMR spectra were recorded on a Bruker Avance III 300 MHz spectrometer. All NMR spectra were referenced on the solvent peak. High resolution mass spectra were obtained using an Agilent 6520 Q-TOF mass spectrometer with an ESI source and an Agilent G1607A coaxial sprayer. All analyses were made in the positive ionization mode. Purine (exact mass for [M+H]^+^ = 121.050873) and 1,2,3,4,5,6-hexakis(2,2,3,3-tetrafluoropropoxy)-1,3,5,2,4,6-triazatriphosphinane (exact mass for [M+H]^+^ = 922.009798) were used for internal mass calibration. IR spectra were recorded on a Shimadzu IR Affinity-1 Fourier Transform infrared spectrometer. Optical rotations were recorded on a Perkin Elmer Polarimeter Model 241 MC. HPLC was performed using a Dionex Summit HPLC system with a Chiralcel OD-H (250 × 4.6 mm, 5 µm) or a Chiralcel OD-R (250 × 4.6 mm, 10 µm) chiral stationary phase. All chemicals were purchased from commercial suppliers and used without further purification unless otherwise stated. All reactions were carried out under inert atmosphere (Ar). Catalysts **3** were prepared as described recently [48,49].

### 3.2. Conditions A: General Procedure for the Phase-Transfer Catalysed Michael-Reaction under Liquid/Solid Phase-Transfer Conditions

Reactions were carried out using 0.2 mmol of the Schiff base **4**. The catalyst **3** (10–20 mol%) and Schiff base **4** (1 eq.) were dissolved in degased mesitylene (0.15 M) and Cs_2_CO_3_ (20 eq.) was added. The vigorously stirred solution (>1200 rpm) was cooled to −20 °C and afterwards the corresponding electrophile (1.5 eq.) was added. After 22 h at −20 °C the reaction mixture was extracted with CH_2_Cl_2_/H_2_O, the combined organic phases were dried over Na_2_SO_4_, evaporated to dryness and purified by column chromatography (silica gel). The Michael-addition products were isolated using heptanes/EtOAc = 40:1 to 10:1 as the eluent.

### 3.3. Conditions B: General Procedure for the Phase-Transfer Catalysed Michael-Reaction under Liquid/Liquid Phase-Transfer Conditions

Reactions were carried out using 0.2 mmol of the Schiff base **4**. The catalyst **3** (20 mol%) and Schiff base **4** (1 eq.) were dissolved in degased toluene (0.15 M) and aqueous RbOH (50%) (25 eq.) was added. The vigorously stirred solution (>1200 rpm) was cooled to −20 °C and afterwards the corresponding electrophile (2 eq.) was added. After 48 h at −20 °C the reaction mixture was extracted with CH_2_Cl_2_/H_2_O, the combined organic phases were dried over Na_2_SO_4_, evaporated to dryness and purified by column chromatography (silica gel). The Michael-addition products were isolated using heptanes/EtOAc = 10:1 as the eluent.

*(S)-(−)-***13a**. Obtained as a colourless oil in 74% yield and with e.r. = 69:31 upon reacting Schiff base **4a** with acrylate **12a** in the presence of 10 mol% catalyst at 0 °C under conditions A. Analytical data are in full accordance with those reported in literature [52,53]. [α]_D_^2^^0^ (c = 0.35, CHCl_3_) = −32.8°; ^1^H-NMR (δ, CDCl_3_, 298 K): 1.44 (s, 9H), 2.17–2.26 (m, 2H), 2.34–2.41 (m, 2H), 3.59 (s, 3H), 3.93–3.99 (m, 1H), 7.14–7.21 (m, 2H), 7.29–7.47 (m, 6H), 7.61–7.68 (m, 2H) ppm; ^13^C-NMR (δ, CDCl_3_, 298 K): 28.0, 28.6, 30.5, 51.5, 64.8, 81.2, 127.8, 128.0, 128.4, 128.6, 128.8, 130.3, 136.5, 139.5, 170.8, 172.9, 173.6 ppm; IR (film): *ῡ*= 2978, 2926, 1738, 1707, 1661, 1599, 1578, 1449, 1369, 1319, 1279, 1260, 1234, 1153, 943, 920, 849, 812 cm^−1^; The enantioselectivity was determined by HPLC (Chiralcel OD-H, eluent: *n*-hexane/*i*-PrOH = 95:5, 0.5 mL/min, 10 °C, retention times: (+)-enantiomer 12.2 min, (−)-enantiomer 15.3 min); HRMS (ESI): *m/z* calcd for C_23_H_27_NO_4_: 382.2013 [M+H]^+^; found: 382.2013. 

*(S)-(−)-*13b. Obtained as a colourless oil in 66% yield and with e.r. = 75:25 upon reacting Schiff base **4b** with acrylate **12a** in the presence of 10 mol% catalyst at 0 °C under conditions A. Analytical data are in full accordance with those reported in literature [54]. [α]_D_^20^ (c = 0.22, CHCl_3_) = −34.4°; ^1^H-NMR (δ, CDCl_3_, 298 K): 2.23–2.32 (m, 2H), 2.33–2.40 (m, 2H), 3.57 (s, 3H), 4.14 (t, *J* = 6.0 Hz, 1H), 5.16 (dd, *J* = 7.0 Hz, 12.5 Hz, 2H), 7.08–7.14 (m, 2H), 7.28–7.42 (m, 11H), 7.59–7.67 (m, 2H) ppm; ^13^C-NMR (δ, CDCl_3_, 298 K): 28.5, 30.4, 51.5, 64.1, 66.6, 127.8, 128.0, 128.1, 128.2, 128.5, 128.7, 128.9, 130.5, 135.8, 136.1, 171.4 (2x), 173.4 ppm; IR (film): *ῡ*= 3063, 2959, 2928, 2853, 1742, 1705, 1659, 1599, 1578, 1499, 1449, 1420, 1389, 1377, 1317, 1279, 1209, 1192, 1177, 1157, 922, 754, 706 cm^−1^; The enantioselectivity was determined by HPLC (Chiralcel OD-H, eluent: *n*-hexane/*i*-PrOH = 99:1, 0.5 mL/min, 10 °C, retention times: (+)-enantiomer 68.4 min, (−)-enantiomer 77.2 min); HRMS (ESI): *m/z* calcd for C_26_H_25_NO_4_: 416.1856 [M+H]^+^; found: 416.1858. 

*(−)-***13c**. Obtained as a colourless oil in 71% yield and with e.r. = 90:10 upon reacting Schiff base **4c** with acrylate **12a** using 20 mol% catalyst at −20 °C under conditions A. Analytical data are in full accordance with those reported in literature [57]. [α]_D_^20^ (c = 0.20, CHCl_3_) = −53.0°; ^1^H-NMR (δ, CDCl_3_, 298 K): 2.20–2.29 (m, 2H), 2.32–2.40 (m, 2H), 3.58 (s, 3H), 3.71 (s, 3H), 4.13 (t, *J* = 6.2 Hz, 1H), 7.14–7.21 (m, 2H), 7.29–7.48 (m, 6H), 7.60–7.67 (m, 2H) ppm; ^13^C-NMR (δ, CDCl_3_, 298 K): 28.6, 30.4, 51.6, 52.2, 64.1, 127.8, 128.1, 128.6, 128.8, 128.9, 130.6, 172.0, 172.2, 173.4 ppm; IR (film): *ῡ*= 3057, 3051, 2992, 2955, 1736, 1624, 1576, 1445, 1437, 1316, 1265, 1204, 1172, 1074, 1028, 1001, 781, 731, 702 cm^−1^; The enantioselectivity was determined by HPLC (Chiralcel OD-H, eluent: *n*-hexane/*i*-PrOH = 99:1, 0.5 mL/min, 10 °C, retention times: (+)-enantiomer 48.5 min, (−)-enantiomer 62.4 min); HRMS (ESI): *m/z* calcd for C_23_H_21_NO_4_: 340.1543 [M+H]^+^; found: 340.1543.

*(−)-***13d**. Obtained as a colourless oil in 68% yield and with e.r. = 89:11 upon reacting Schiff base **4c** with acrylate **12b** using 20 mol% catalyst at −20 °C under conditions A. [α]_D_^20^ (c = 0.67, CHCl_3_) = −50.8°; ^1^H-NMR (δ, CDCl_3_, 298 K): 0.90 (t, *J* = 7.3 Hz, 3H), 1.28–1.38 (m, 2H), 1.48–1.59 (m, 2H), 2.20–2.29 (m, 2H), 2.30–2.38 (m, 2H), 3.71 (s, 3H), 3.98 (t, *J* = 6.7 Hz, 2H), 4.12 (t, *J* = 5.9 Hz, 1H), 7.15–7.21 (m, 2H), 7.29–7.48 (m, 6H), 7.61–7.67 (m, 2H) ppm; ^13^C-NMR (δ, CDCl_3_, 298 K): 13.7, 19.1, 28.6, 30.6, 30.6, 52.2, 64.2, 64.4, 127.8, 128.1, 128.6, 128.8, 128.9, 130.5, 136.1, 139.3, 171.2, 172.2, 173.0 ppm; IR (film): *ῡ*= 3057, 2957, 2934, 2872, 1732, 1661, 1624, 1597, 1578, 1491, 1447, 1437, 1393, 1364, 1317, 1265, 1204, 1175, 1074, 1028, 1001, 941, 920, 781, 764, 737 cm^−1^; The enantioselectivity was determined by HPLC (Chiralcel OD-H, eluent: *n*-hexane/*i*-PrOH = 99:1, 0.5 mL/min, 10 °C, retention times: (+)-enantiomer 23.4 min, (−)-enantiomer 24.6 min); HRMS (ESI): *m/z* calcd for C_23_H_27_NO_4_: 382.2013 [M+H]^+^; found: 382.2015.

*(−)-***13f**. Obtained as a colourless oil in 81% yield and with e.r. = 87:13 upon reacting Schiff base **4c** with acrylate **12d** using 20 mol% catalyst at −20 °C under conditions A. [α]_D_^20^ (c = 0.63, CHCl_3_) = −45.9°; ^1^H-NMR (δ, CDCl_3_, 298 K): 2.23–2.32 (m, 2H), 2.38–2.45 (m, 2H), 3.71 (s, 3H), 4.14 (t, *J* = 6.0 Hz, 1H), 5.02 (s, 2H), 7.13–7.19 (m, 2H), 7.28–7.46 (m, 11H), 7.61–7.67 (m, 2H) ppm; ^13^C-NMR (δ, CDCl_3_, 298 K): 28.6, 30.6, 52.2, 64.1, 66.3, 127.8, 128.1, 128.2, 128.5, 128.6, 128.8, 128.9, 130.6, 135.9, 136.1, 172.1, 172.8 ppm; IR (film): *ῡ*= 3059, 3036, 2951, 1732, 1659, 1622, 1597, 1578, 1491, 1447, 1420, 1385, 1316, 1265, 1206, 1159, 1074, 1028, 1001, 988, 974, 962, 943, 922, 912, 847, 735 cm^−1^; The enantioselectivity was determined by HPLC (Chiralcel OD-H, eluent: *n*-hexane/*i*-PrOH = 99:1, 0.5 mL/min, 10 °C, retention times: (+)-enantiomer 76.3 min, (−)-enantiomer 83.9 min); HRMS (ESI): *m/z* calcd for C_26_H_25_NO_4_: 416.1856 [M+H]^+^; found: 416.1861. 

*(−)-***16a**. Obtained as a colourless oil in 65% yield and with e.r. = 75:25 upon reacting Schiff base **4a** with acrylamide **14a** under conditions B. [α]_D_^20^ (c = 0.24, CHCl_3_) = −15.8°; ^1^H-NMR (δ, CDCl_3_, 298 K): 0.80 (s, 9H), 2.06–2.20 (m, 2H), 2.21–2.43 (m, 2H), 3.93 (dd, *J* = 5.6, 6.35 Hz, 1H), 7.04–7.13(m, 2H), 7.17-7.40 (m, 6H), 7.52–7.72 (m, 2H) ppm; ^13^C-NMR (δ, CDCl_3_, 298 K): 28.1, 29.3, 29.6, 35.4, 37.3, 65.0, 81.1, 127.7, 128.0, 128.5, 128.6, 128.8, 130.3, 136.5, 139.6, 170.4, 171.2, 172.6 ppm; IR (film): *ῡ*= 2960, 2880, 2560, 1720, 1640, 1520, 1440, 1400, 1360, 1320, 1280, 1160, 1080, 880 cm^−1^; The enantioselectivity was determined by HPLC (Chiralcel OD-R, eluent: AcN*/*H_2_O = 55:45, 0.7 mL/min, 10 °C, retention times: (+)-enantiomer 11.6 min, (−)-enantiomer 13.1 min); HRMS (ESI): *m/z* calcd for C_24_H_30_N_2_O_3_: 395.2329 [M+H]^+^; found: 395.2325. 

*S*-*(−)-***17**. Obtained as a colourless oil in 97% yield and with e.r. = 77:23 upon reacting Schiff base **4c** with MVK (**15**) using 20 mol% catalyst at −20 °C under conditions A. [α]_D_^20^ (c = 0.46, CHCl_3_) = −34.1°; ^1^H-NMR (δ, CDCl_3_, 298 K): 2.11 (s, 3H), 2.13–2.21 (m, 2H), 2.48–2.55 (m, 2H), 3.71 (s, 3H), 4.11 (t, *J* = 6.1 Hz, 1H), 7.14–7.19 (m, 2H), 7.30–7.48 (m, 6H), 7.61–7.66 (m, 2H) ppm; ^13^C-NMR (δ, CDCl_3_, 298 K): 27.6, 29.9, 39.7, 52.2, 64.0, 127.7, 128.1, 128.6, 128.8, 128.9, 130.5, 136.1, 139.3, 172.4 (2×), 208.0 ppm; IR (film): *ῡ*= 3080, 3055, 2953, 2930, 2173, 1736, 1714, 1659, 1622, 1599, 1578, 1491, 1447, 1437, 1358, 1317, 1275, 1265, 1204, 1177, 1161, 1094, 1074, 1042, 1028, 1001, 941, 920, 810, 783, 766, 733, 698 cm^−1^; The enantioselectivity was determined by HPLC (Chiralcel OD-H, eluent: *n*-hexane/*i*-PrOH = 99:1, 0.5 mL/min, 10 °C, retention times: (+)-enantiomer 69.0 min, (−)-enantiomer 84.7 min); HRMS (ESI): *m/z* calcd for C_20_H_21_NO_3_: 324.1594 [M+H]^+^; found: 324.1593.

## 4. Conclusions

Summarizing, the herein developed late stage acetal-transformation strategy did not allow us to obtain novel catalysts **3** in a reliable and straightforward fashion especially due to problems associated with the catalyst counter anion and the hereby formed hardly removable impurities. To get a detailed understanding of the application scope and limitations of our catalysts we tested them in a variety of different important transformations and found that, although these compounds have recently shown their good potential in the asymmetric α-alkylation of glycine Schiff bases, they clearly failed when we attempted to control more reactive nucleophiles like β-keto esters. On the other hand using them to catalyse the Michael addition of glycine Schiff bases to different acceptors very interesting results have been obtained. It was found necessary to carefully optimize the reaction conditions for every single substrate class, as seemingly small structural changes required the use of totally different reaction conditions. Unfortunately, the strikingly different behaviour of different nucleophiles and different electrophiles and also the need for totally different reaction conditions compared to the standard alkylation reaction is not fully understood yet. This highlights again the necessity of carrying out careful screening studies and the problems of transferring knowledge gathered in one test system to another one, especially in complex heterogeneous reaction systems as usually employed in asymmetric phase-transfer catalysis. In addition, we observed again a very strong influence of the counter anions on the catalyst performance, thus making a strict control of the anion necessary. Under carefully optimized conditions enantiomeric ratios of up to 91:9 could be achieved in the addition of glycine Schiff bases to acrylates whereas acrylamides and methyl vinyl ketone were less well tolerated (up to e.r. 77:23 in these cases). Accordingly, we have now a rather detailed understanding about the scope and limitations of the synthesis sequence to access our PTCs and about the application scope of these catalysts in asymmetric transformations. 

## Supplementary Materials

Supplementary materials can be accessed at: http://www.mdpi.com/1420-3049/18/4/4357/s1.
