# Supplementary material for: Application Scope and Limitations of TADDOL-Derived Chiral Ammonium Salt Phase-Transfer Catalysts
Source: Molecules. 2013 Apr 12;18(4):4357–72. doi: 10.3390/molecules18044357 (PMC4202194; doi:10.3390/molecules18044357)
Supplement: Supplementary file 1 [file molecules-18-04357-s001.pdf]

Supporting Information – HPLC

Operator:admin Timebase:Summit_1 Sequence:WAS_190112

19.1.2012 12:08 PM

2 HER-489-01

Sample Name:	**HER-489-01**	*Injection Volume:*	**10,0**
Vial Number:	**RA2**	*Channel:*	**UV_VIS_2**
Sample Type:	**unknown**	*Wavelength:*	**n.a.**
Control Program:	**TEST_30**	*Bandwidth:*	**n.a.**
Quantif. Method:	**default**	*Dilution Factor:*	**1,0000**
Recording Time:	**19.1.2012 11:35**	*Sample Weight:*	**1,0000**
Run Time (min):	**30,00**	*Flow ml/min:*	**0,5**

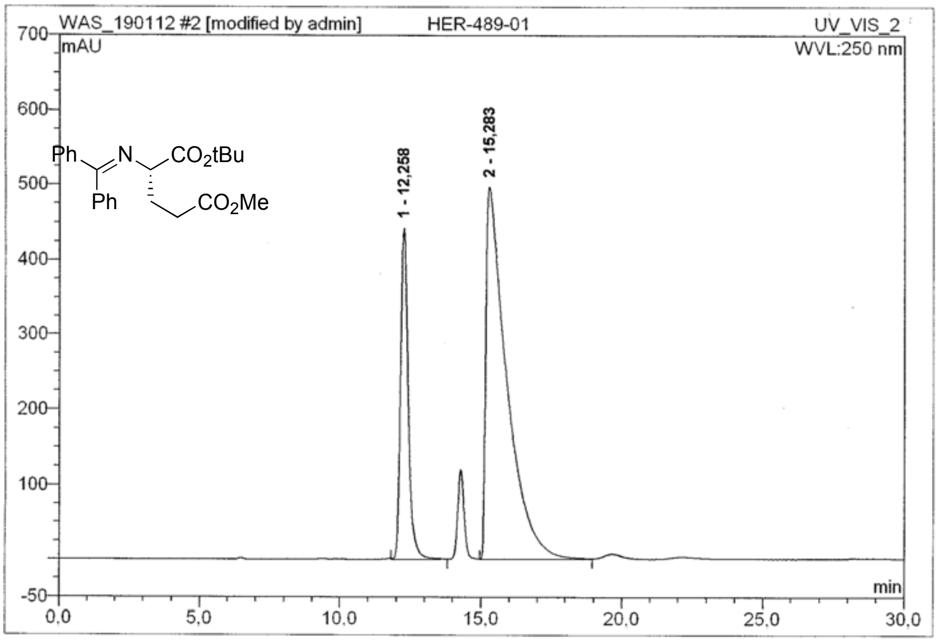

No.	Ret.Time min	Peak Name	Height mAU	Area mAU*min	Rel.Area %	Amount	Type
1	12,26	n.a.	441,651	138,802	24,58	n.a.	M *
2	15,28	n.a.	496,818	425,930	75,42	n.a.	M *
**Total:**			938,468	564,732	100,00	0,000	

Rueckl/Integration

Chromeleon (c) Dionex 1996-2006
Version 6.80 SR10 Build 2818 (166959)

Operator:admin Timebase:Summit_1 Sequence:WAS_131212 Page 1-1
13.12.2012 12:13 PM

2 HER-920-02 EX-145

Sample Name:	**HER-920-02**	*Injection Volume:*	**10,0**
Vial Number:	**RA2**	*Channel:*	**UV_VIS_2**
Sample Type:	**unknown**	*Wavelength:*	**n.a.**
Control Program:	**NP_99-1_120min**	*Bandwidth:*	**n.a.**
Quantif. Method:	**default**	*Dilution Factor:*	**1,0000**
Recording Time:	**13.12.2012 10:17**	*Sample Weight:*	**1,0000**
Run Time (min):	**112,41**	*Flow ml/min:*	**0,50**

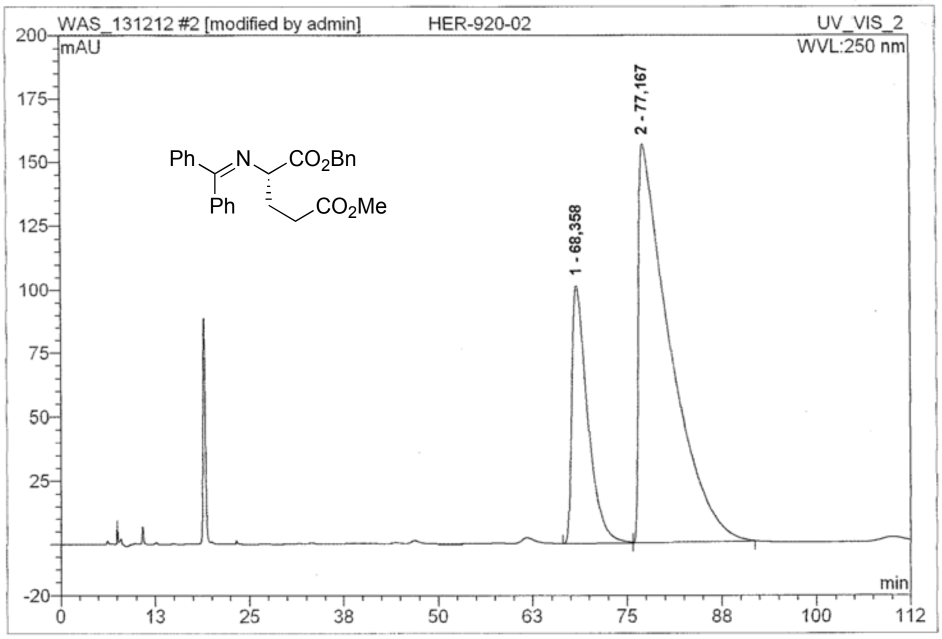

No.	Ret.Time min	Peak Name	Height mAU	Area mAU*min	Rel.Area %	Amount	Type
1	68,36	n.a.	101,397	245,748	24,70	n.a.	BM
2	77,17	n.a.	156,272	749,321	75,30	n.a.	MB
Total:			257,669	995,070	100,00	0,000	

Rueckl/Integration

Chromeleon (c) Dionex 1996-2006
Version 6.80 SR10 Build 2818 (166959)

Operator:admin Timebase:Summit_1 Sequence:WAS_310113

Page 1-1
31.1.2013 2:53 PM

3 HER-973-2 EX-144

Sample Name:	**HER-973-2**	*Injection Volume:*	**10,0**
Vial Number:	**RA3**	*Channel:*	**UV_VIS_1**
Sample Type:	**unknown**	*Wavelength:*	**n.a.**
Control Program:	**NP_99-1_90min**	*Bandwidth:*	**n.a.**
Quantif. Method:	**default**	*Dilution Factor:*	**1,0000**
Recording Time:	**31.1.2013 12:51**	*Sample Weight:*	**1,0000**
Run Time (min):	**90,00**	*Flow ml/min:*	**0,50**

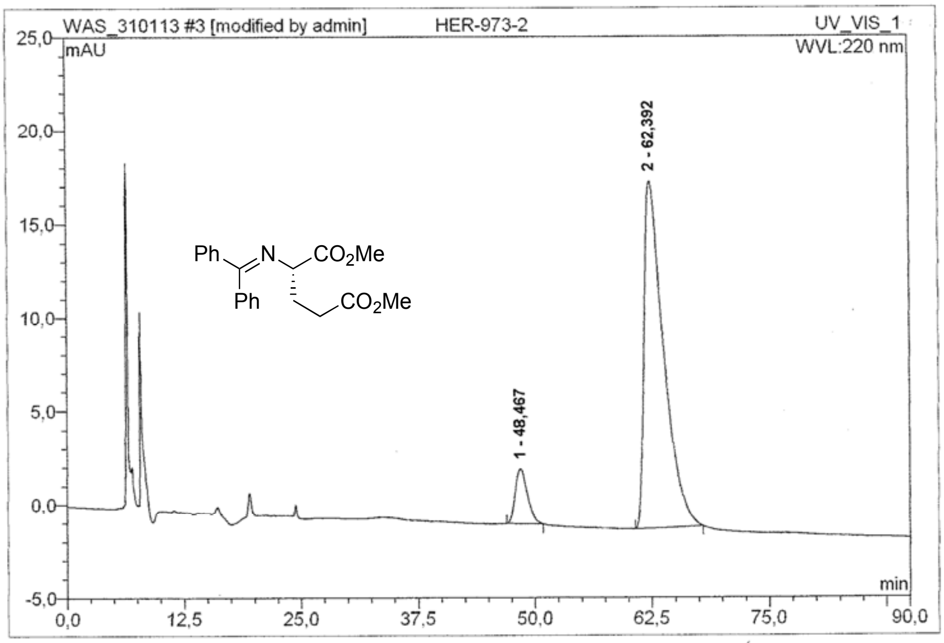

No.	Ret.Time min	Peak Name	Height mAU	Area mAU*min	Rel.Area %	Amount	Type
1	48,47	n.a.	2,939	4,442	9,05	n.a.	BMB
2	62,39	n.a.	18,546	44,615	90,95	n.a.	BMB
**Total:**			21,485	49,057	100,00	0,000	

Rueckl/Integration

Chromeleon (c) Dionex 1996-2006
Version 6.80 SR10 Build 2818 (166959)

Operator:admin Timebase:Summit_1 Sequence:WAS_170113 Page 1-1
17.1.2013 12:04 PM

1 HER-956-2 Ex-152

Sample Name:	**HER-956-2**	*Injection Volume:*	**10,0**
Vial Number:	**RA1**	*Channel:*	**UV_VIS_2**
Sample Type:	**unknown**	*Wavelength:*	**n.a.**
Control Program:	**NP_99-1_90min**	*Bandwidth:*	**n.a.**
Quantif. Method:	**default**	*Dilution Factor:*	**1,0000**
Recording Time:	**17.1.2013 10:48**	*Sample Weight:*	**1,0000**
Run Time (min):	**59,10**	*Flow ml/min:*	**0,50**

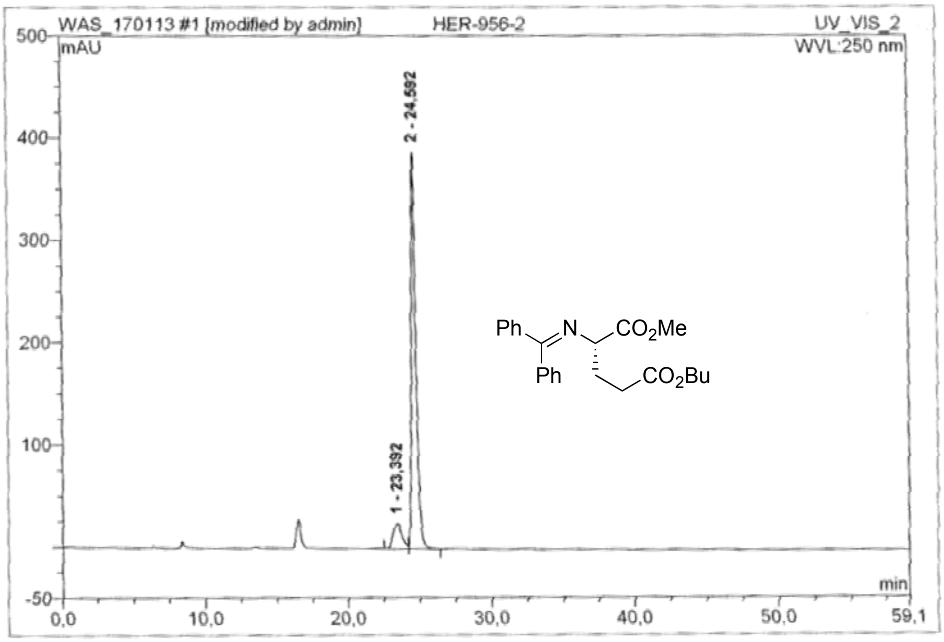

No.	Ret.Time min	Peak Name	Height mAU	Area mAU*min	Rel.Area %	Amount	Type
1	23,39	n.a.	24,364	17,606	10,90	n.a.	BM
2	24,59	n.a.	387,931	143,944	89,10	n.a.	MB
**Total:**			412,295	161,550	100,00	0,000	

Rueckl/Integration

Chromeleon (c) Dionex 1996-2006
Version 6.80 SR10 Build 2818 (166959)

Operator:admin Timebase:Summit_1 Sequence:WAS_010213

Page 1-1
1.2.2013 12:56 PM

2 HER-979-2 , EX-160

Sample Name:	**HER-979-2**	*Injection Volume:*	**10,0**
Vial Number:	**RA4**	*Channel:*	**UV_VIS_2**
Sample Type:	**unknown**	*Wavelength:*	**n.a.**
Control Program:	**NP_99-1_120min**	*Bandwidth:*	**n.a.**
Quantif. Method:	**default**	*Dilution Factor:*	**1,0000**
Recording Time:	**1.2.2013 10:57**	*Sample Weight:*	**1,0000**
Run Time (min):	**118,17**	*Flow ml/min:*	**0,50**

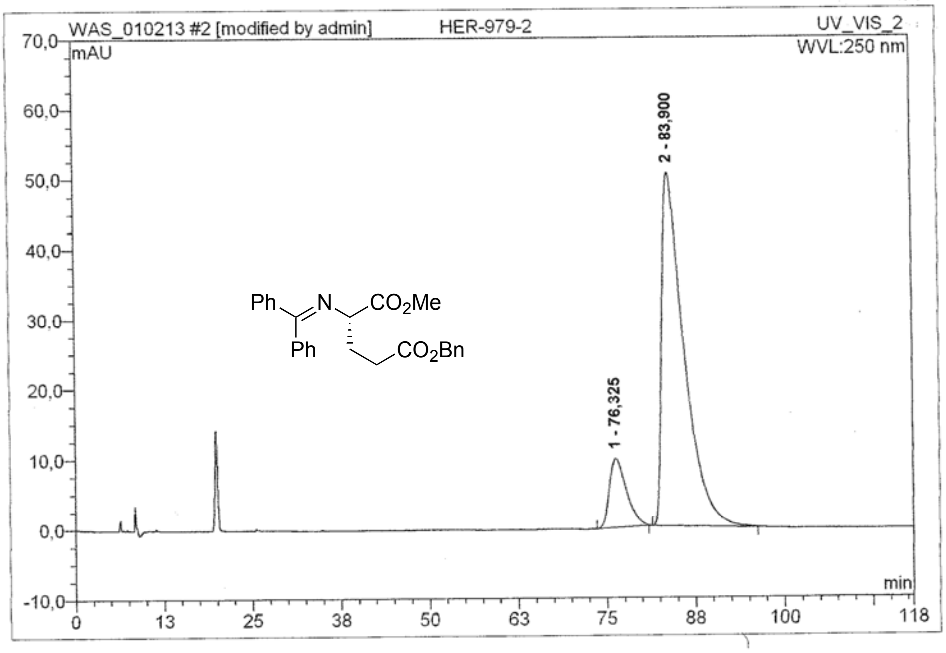

No.	Ret.Time min	Peak Name	Height mAU	Area mAU*min	Rel.Area %	Amount	Type
1	76,33	n.a.	9,859	27,520	13,13	n.a.	BMB*
2	83,90	n.a.	50,412	182,137	86,87	n.a.	BMB*
**Total:**			60,271	209,657	100,00	0,000	

Rueckl/Integration

Chromeleon (c) Dionex 1996-2006
Version 6.80 SR10 Build 2818 (166959)

Operator:admin Timebase:Summit_1 Sequence:WAS_151012 Page 1-1
6.2.2013 2:24 PM

4 GN-151-03

Sample Name:	**GN-151-03**	*Injection Volume:*	**10,0**
Vial Number:	**RA4**	*Channel:*	**UV_VIS_2**
Sample Type:	**unknown**	*Wavelength:*	**n.a.**
Control Program:	**OD_R_40_5**	*Bandwidth:*	**n.a.**
Quantif. Method:	**default**	*Dilution Factor:*	**1,0000**
Recording Time:	**15.10.2012 14:43**	*Sample Weight:*	**1,0000**
Run Time (min):	**20,12**	*Flow ml/min:*	**0,70**

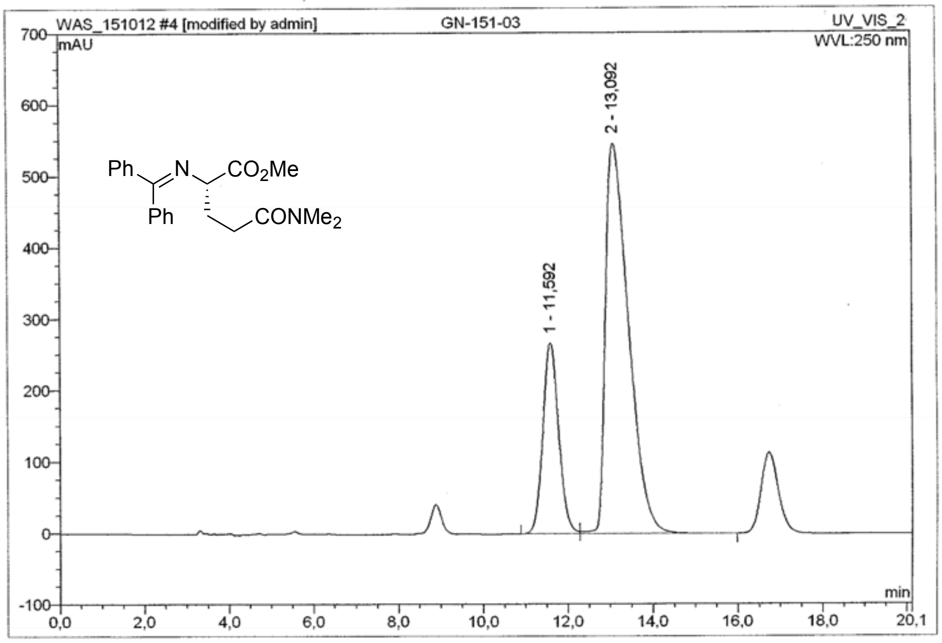

No.	Ret.Time min	Peak Name	Height mAU	Area mAU*min	Rel.Area %	Amount	Type
1	11,59	n.a.	266,407	115,647	25,22	n.a.	BM *
2	13,09	n.a.	545,676	342,868	74,78	n.a.	MB*
**Total:**			812,083	458,515	100,00	0,000	

45 Vol% H2O
55 Vol% Acetonitril

Rueckl/Integration

Chromeleon (c) Dionex 1996-2006
Version 6.80 SR10 Build 2818 (166959)

Operator:admin Timebase:Summit_1 Sequence:WAS_010213

Page 1-1
1.2.2013 11:08 AM

1 HER-982-2 EX-161

Sample Name:	**HER-982-2**	*Injection Volume:*	**10,0**
Vial Number:	**RA5**	*Channel:*	**UV_VIS_1**
Sample Type:	**unknown**	*Wavelength:*	**n.a.**
Control Program:	**NP_99-1_120min**	*Bandwidth:*	**n.a.**
Quantif. Method:	**default**	*Dilution Factor:*	**1,0000**
Recording Time:	**1.2.2013 8:55**	*Sample Weight:*	**1,0000**
Run Time (min):	**120,00**	*Flow ml/min:*	**0,50**

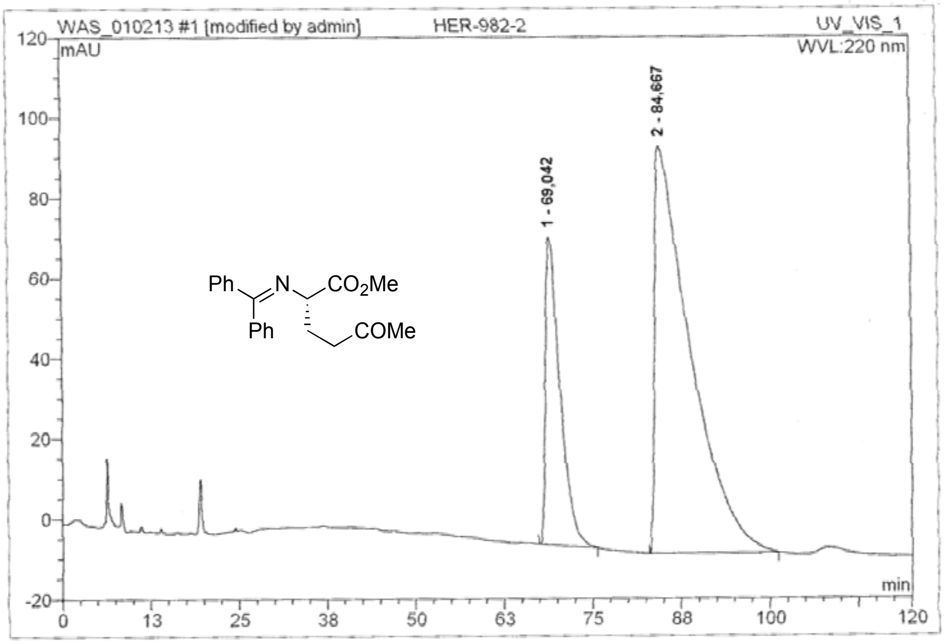

No.	Ret.Time min	Peak Name	Height mAU	Area mAU*min	Rel.Area %	Amount	Type
1	69,04	n.a.	76,534	180,467	23,51	n.a.	BMB
2	84,67	n.a.	101,355	587,012	76,49	n.a.	BMB
**Total:**			177,889	767,480	100,00	0,000	

Rueckl/Integration

Chromeleon (c) Dionex 1996-2006
Version 6.80 SR10 Build 2818 (166959)
